# Quantifying Nature’s Bistability: Simulation of Earwig Fan Folding

**DOI:** 10.3390/biomimetics11010009

**Published:** 2025-12-24

**Authors:** Nele Binder, Leone Costi, Dario Izzo, Tobias Seidl

**Affiliations:** 1 Westfälisches Institut für Bionik, Westfälische Hochschule, 46397 Bocholt, Germany; tobias.seidl@w-hs.de; 2 Advanced Concepts Team, European Space Research and Technology Centre, European Space Agency, 2201 AZ Noordwijk, The Netherlands

**Keywords:** biomechanics, *Dermaptera*, earwig, wing folding, bistability, MuJoCo

## Abstract

In this work, a numerical tool is presented to simulate the dynamics of insect wing folding by example of the fan folding of the dermapteran hindwing. The scalability of the system is demonstrated by generalising the mechanical behaviour from the small geometry of the wing to a suitable scale for engineering applications, such as deployable structures for space applications. The tool is written in Python and based on the MuJoCo physics engine. Sections of the anal fan are modelled as a bar-and-hinge model with elastic tendons, allowing a high number of design parameters and fast computation. In light of these advantages, the wing folding and unfolding behaviour is investigated with respect to the tendon’s elastic properties and the actuation of the deformation. Bistability is characterised using a single tendon and the entire fan section. Given the upscaled geometry of the analysed section, the required tendon characteristics to transition between the stable states are identified within a reasonable range for technological transfer towards biomimetic structures modelled after the dermapteran hindwing.

## 1. Introduction

The dermapteran hindwing is known for its remarkable folding ratio of 1:10 in the European earwig (*Forficula auricularia*) or 1:18 in the lesser earwig (*Labia minor*) [1,2]. Moreover, the dermapteran hindwing displays a bistable behaviour, switching between the unfolded and folded state without internal musculature present in the wing [3,4]. Such a mechanical system is very desirable in lightweight engineering solutions, aiming to reduce mass for functional and economic reasons, while maintaining stiffness and reliability [5]. This desire is also evident in the development of deployable structures in the aerospace industry [6,7], where solutions are increasingly inspired by nature. This includes the dermapteran hindwing, due to its complex folding pattern, self-stabilising mechanisms, and minimal actuation [8,9,10]. In order to optimise the design of deployable structures and develop biomimetic folding systems, it is important to understand the mechanisms that underlie the large deformation of the dermapteran hindwing. In the following, these mechanisms and previous modelling approaches are reviewed to map the current state of modelling insect wing folding. This provides the foundation for a new modelling approach, aimed at bridging the gap between biological accuracy and technical application.

**Figure 1:**
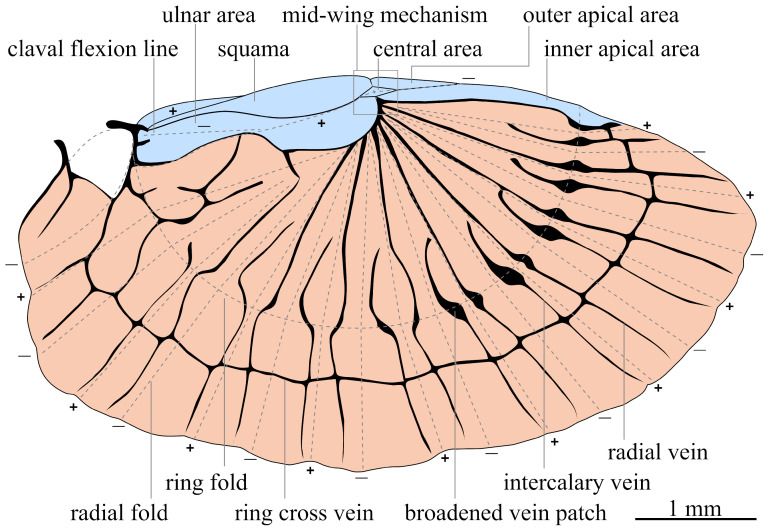
Schematic drawing of the dermapteran hindwing, modified after [1,2]. The marginal area is highlighted in blue, and the anal fan in orange. Dashed lines indicate fold lines, whereas + marks a mountain fold and − marks a valley fold.

The folding pattern of the dermapteran hindwing can be divided into two areas: the marginal area, including the mid-wing mechanism, and the anal fan area (Figure 1). The mid-wing mechanism stands out as one of the best-understood mechanisms of the complex dermapteran wing. It consists of the squama, ulnary area, inner and outer apical area, creating four fold-lines meeting in the central elastic area [1,3]. This mechanism is a basic, single-node model with one degree of freedom (DoF) common in insect wing folding [11,12]. As observed in *Dermaptera*, *Coleoptera*, and *Blattodea*, the kinematics of the simplified four-plate mechanism is modelled analytically, describing deformation and velocity during the folding process [4,13]. Furthermore, based on the four-plate mechanism in *Coleoptera*, an analytical and numerical model using ADAMS is created to simulate folding dynamics using motorised torsion hinges to model the fold lines [14].

Bistability is defined as two local potential energy minima, where the system remains stable in one of the minima until energy is provided to switch the stable state [15,16]. In the dermapteran hindwing, the mid-wing mechanism is bistable. The bistability results from local elasticity in the central area, due to the presence of resilin, a protein capable of storing elastic energy in the unfolded wing [17,18,19,20]. The approximate Young’s modulus of resilin is documented ranging from 0.4 MPa to 1.5 MPa [21]. Furthermore, the sum of all angles between the plates results in 350°, leading to a pyramid shape, which is not stable in the fully flat configuration. Studies dedicated towards self-folding, bistable origami are inspired by this mechanism. Faber et al. model the creases analytically and numerically in ABAQUS/Explicit with a combination of extensional and rotational springs, introducing a second DoF [16]. Thereby, the choice of the solver allows optimising towards convergence and computational time [16,22]. Their models are validated by building a physical model using a pre-stretched membrane as creases between rigid plates [16]. Rojas et al. introduce programmable pre-strain using bi-layer creases made of shape memory polymers. They modelled this compliant origami analytically and numerically in ABAQUS/Standard as a static analysis, but with non-linear effects enabled to allow large deformation [23]. Both models are validated with a physical model [23]. By altering the shape and position of the stiff plates and elastic creases, Han et al. demonstrate a bio-inspired multi-stable origami with three degrees of freedom [24]. The non-linear dynamic system is modelled geometrically with Lagrange’s Equations compared to numerical simulations in ADAMS, using a combination of extensional and rotational springs to model the soft creases. Their model displays six different stable states, which are validated by a physical model [24].

Contrary to the mid-wing mechanism, the dynamics of the anal fan folding are less comprehensively analysed. The fan folding is composed of the radial fold lines and the transversal ring fold. The high folding ratio of the fan is achieved, as multiple simple-nodes are stacked to multi-node foldings [12,13,25,26,27]. The ring fold is crossing broadened vein patches of the alternating radial and intercalary veins, which contain resilin. Two types of broadened vein patches can be distinguished. In radial veins, they are described as closed patches, with resilin being present on the dorsal side of the wing. In intercalary veins, the resilin is found on the ventral side of the so-called open patches [1,27,28,29]. With the resilin placement correlating to different bending directions, the correct folding configuration is maintained and incorrect folding is prevented [1,29]. The energy in the patches is released by the thoracic musculature, causing movement in the wing articulation. The unfolding of the wing is observed to be either happening by pulling the wing open with the cerci or by shaking it, depending on the species [12,30,31,32]. With the help of the cerci, the rotation of the mid-wing is initiated, transmitting the force to the radial veins through the basal vein coupling [1,4].

The geometry of the fan folding pattern is described and parametrised as an algorithm by Saito et al. [27]. Their algorithm is informed by µ-CT footage and validated by reconstructing wing geometries of dermapteran fossils. The deformation of the folding pattern is demonstrated using the online tool Origami Simulator [27]. This simulator was developed to numerically compute the folded state of origami patterns at high speed, using compliant constraints [33]. Whilst it correctly visualises axial strain based on the deformation, it does not capture the physical behaviour. Therefore, it does not consider kinematics like rigid origami methods [34,35,36], local structural behaviour like finite element analysis [37,38,39], or, lastly, bar-and-hinge models capture mechanics while focusing on efficient abstraction and computation [40,41,42].

However, any physical model, built based on the algorithm of Saito et al., with a thickness greater than that of paper, is not foldable. In alignment with the functionality of the Origami Simulator, their algorithm is based on a zero-thickness approach. As a possible solution, Kitajima et al. propose an extension of the algorithm that adjusts the folding pattern to consider thickness and enables building of foldable, kinematic models [43,44]. Still, the dermapteran fan folding is not yet modelled as a dynamic system, using an analytical or numerical approach.

Regardless of the mechanism in question, physical, analytical, and numerical methods are used to model the large deformations during insect wing folding. Physical models have advantages and disadvantages stemming from their simplicity. While these models do not properly mirror the complexity of an insect wing, they are quick to manufacture and demonstrate simple deformations, visually helping in understanding the mechanisms [45,46]. On one hand, physical models are said to have a bias, as “they are built to work” and require research on the biological wing to validate the physical model [46]. On the other hand, physical models are suitable for validating mathematical models. Mathematical models can be analytical or numerical. Analytical models require a simplified wing geometry but allow quick predictions for selected, altered parameters. In comparison, numerical models, for example, finite element analysis (FEA), allow for more complex modelling of biological systems. However, defining geometry, boundary, and loading conditions is time-consuming, particularly if high accuracy is desired for the model [45,46]. Although FEA is the most accurate modelling method accessible, the results can still differ from real insect wings. This can happen if the model is not detailed enough regarding the complex geometry and anisotropic material properties [46,47,48]. For example, Jin et al. compare a FE model and a real beetle wing in a bending test, demonstrating that minor simplifications in the cross-sectional geometry can lead to inaccurate results [49]. Herbert et al. model the cambering of the hindwing of the desert locust during flight in ANSYS [50]. They validate their most detailed models, while in less accurate models, the deformations differ qualitatively from the in vivo studies. However, both FE models of the beetle and locust wing address only small deformations, whereas the modelling of wing folding as large complex deformations is not realisable using FE models due to their complexity and high computational demand [50].

In the aim of reducing the time costs of creating complex but accurate models of insect wings and automating this process, the tools WingMesh and WingGram were developed for Matlab [47,48]. WingMesh is based on Distmesh2 and allows out-of-plane meshing of insect wings. However, the type and size of the generated mesh can not be controlled, leading to the improved modelling tool WingGram for the mechanical analysis of insect wings. WingGram extracts the wing geometry out of a 2D image, which is then translated into a mesh and manipulatable with the help of Python scripting in ABAQUS. By enabling faster and more precise meshing, the process for numerical biomechanical studies is accelerated, which is expected to be especially advantageous for comparative studies [47,48]. However, while the tool is already used in aerodynamic modelling of insect wings [51], no simulation work on wing deformation or folding is found to use WingGram yet.

We believe that modelling of biomechanical systems can offer a greater understanding of how nature solves complex mechanical challenges and enables transfer to technological applications. However, in the case of insect wing folding, an absence of numerical tools that allow fast modelling and simulation is noticeable. In turn, this led to a lack of comprehensive studies of multiple varying parameters to test scalability and enable transfer to technological applications. To counteract this, a simulation tool is presented here, developed in Python, based on the open source physics engine MuJoCo [52].

The fan contributes the most to the efficient folding ratio, by a factor of five to nine [53]. Still, its functional morphology, including the coupling of the basal vein and broadened vein patches, remains less well studied and understood than the mid-wing mechanism. With the help of our tool, the folding and unfolding of one section of the anal fan in the dermapteran hindwing is studied. The dynamic system is modelled as a rigid foldable origami with no compliant creases, while using elastic tendons on a mass-loaded bar-and-hinge model to capture the bistability of the hindwing’s folding pattern and anisotropic material distribution. For this, an upscaled wing geometry is used to reconstruct the transition between the unfolded and folded state, based on a range of stiffnesses and torques. By conducting a parameter search, predictions are made under which conditions the transfer of this dynamic system to real-world applications is feasible. To demonstrate the concept, a bistable hinge using accessible additive manufacturing techniques is designed. For this purpose, the mechanical properties of a commonly used elastic material are characterised and set in the context of the simulation tool, determining whether it can be used to assist in the design process of bistable deployable structures in the future.

## 2. Materials and Methods

The simulation tool is written in Python 3.10.15 (Python Software Foundation, Beaverton, OR, USA) [54] using MuJoCo 3.2.0 (DeepMind Technologies Ltd., London, UK) [52]. The solution is integrated using the semi-implicit Euler method:

(1) $$v_{t+h}=v_{t}+h\cdot a_{t}$$

(2) $$q_{t+h}=q_{t}+h\cdot v_{t+h}$$

where the joint velocity $v_{t+h}$ is calculated as the sum of the velocity of the previous step $v_{t}$ and the acceleration $a_{t}$ multiplied by the timestep width *h*. The new value of the velocity $v_{t+h}$ is then multiplied by *h* and added to the previous joint position $q_{t}$ to calculate the new joint position $q_{t+h}$ [55]. The timestep width *h* set for the wing folding simulations is 0.005 s.

**Figure 2:**
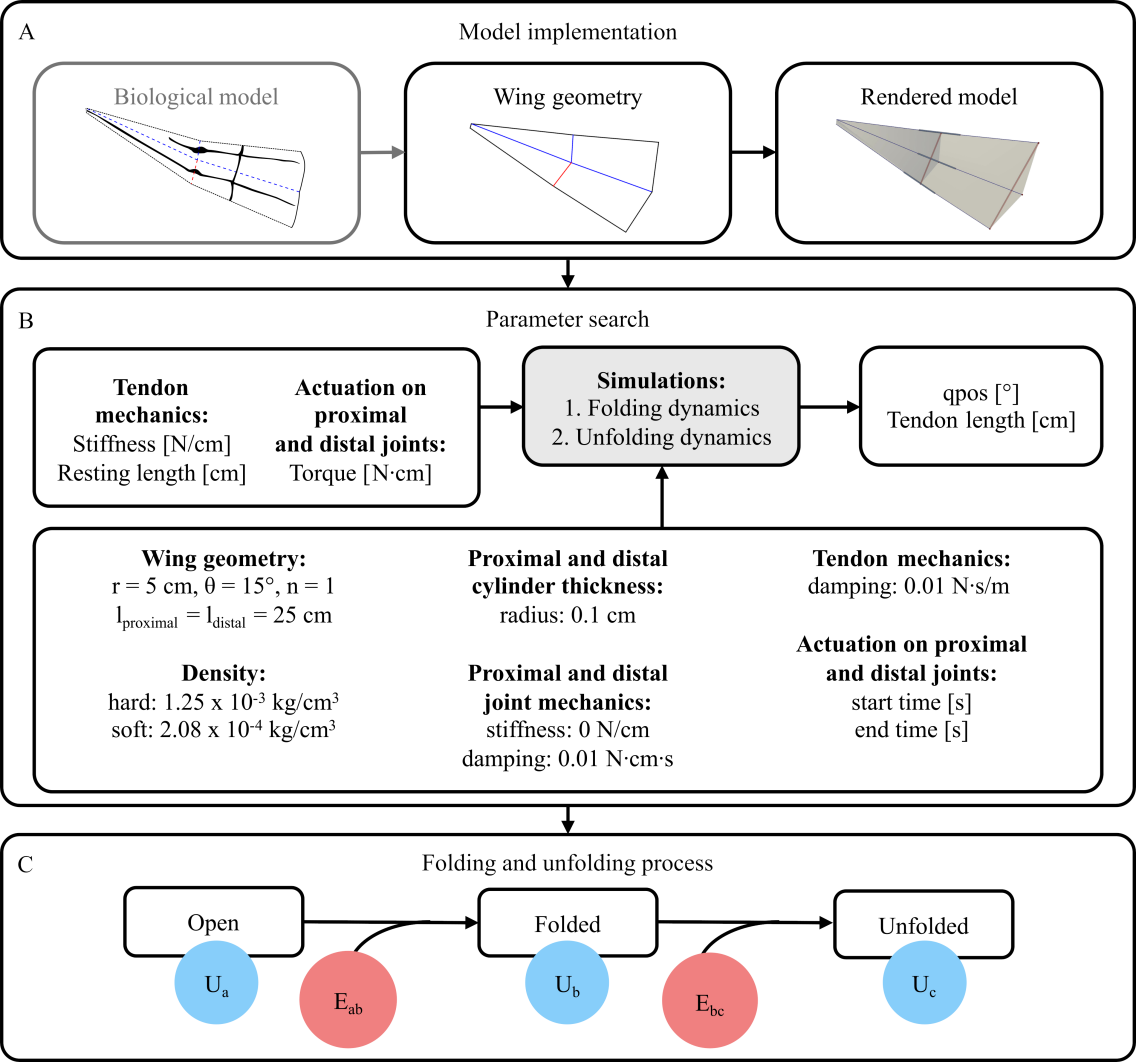
Flowchart of the protocol for simulating the dynamic system of a fan section modelled after the dermapteran hindwing for scaling purposes. Based on the biological model, the geometry of the fan section is generated and turned into an XML model (**A**). A parameter search is conducted (**B**), analysing the energetic landscape, characterised by the energies at the respective state $U_{a}$–$U_{c}$, and the with the input energies $E_{ab}$ and $E_{bc}$ to transition between states (**C**).

The general process for simulating and analysing insect wing folding, specifically using MuJoCo, is depicted in Figure 2. In summary, a foldable geometric pattern of the wing is required, for which a model in extensible markup language (XML) is generated that considers local elasticities in the wing. Using a geometrically constant wing model, a parameter search on behalf of the elastic force and torque-driven components is conducted, gathering insights on the scalability of the dynamic system folding and unfolding.

To ensure foldability of dermapteran wing geometries of any scale, the folding pattern is generated based on the algorithm of Saito et al. [27]. This algorithm is simplified to reduce the number of input parameters down to four: the desired length of the proximal and distal segments, $l_{proximal}$ and $l_{distal}$, the radius *r* and θ for the angular width of the segments, to deflect the fold lines by $\phi_{i}$. For the amount *n* of fan units, the starting points and vectors of 2n+3 fold lines need to be calculated to properly compute the folding angles in the final simulation model with 2n+1 fold lines. In consideration of the simplifications made to the Saito-algorithm, the flat-foldability is given for $n\cdot \theta \leq 90^{\circ }$.

**Figure 3:**
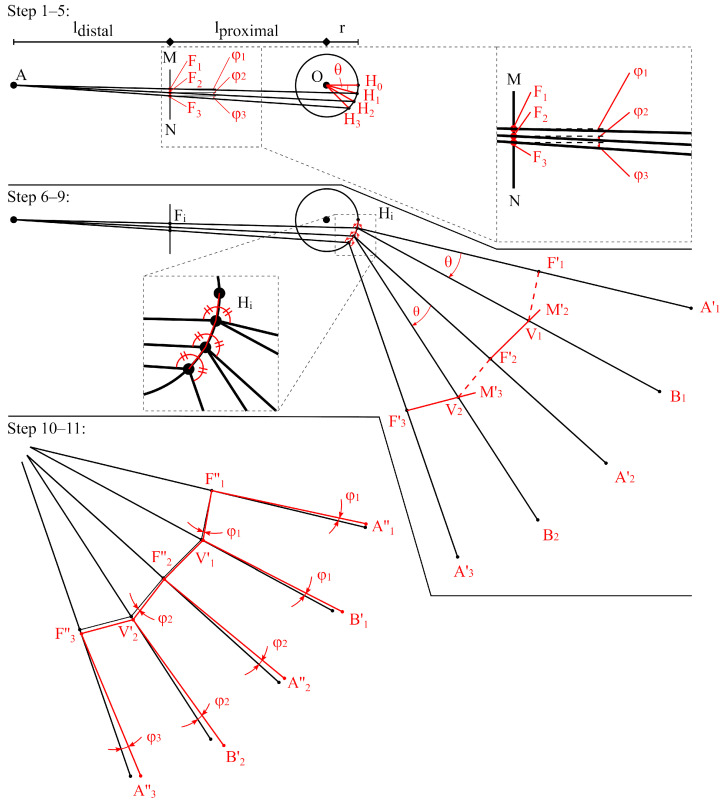
Visual explanation of the algorithm used for earwig fan folding patterns, illustrated for the geometry of a wing model with n=1. The parameters $l_{proximal}$, $l_{distal}$, *r*, and θ define the folded geometry (step 1–5), of which the unfolded geometry is generated step by step (step 6–11).

The algorithmic process is illustrated in Figure 3. First, the centre of the fan *O* is defined by a circle of radius *r*. Point *A* lies horizontally from *O*, at a distance equal to the sum of proximal and distal lengths. A vertical line M−N is placed at the proximal length from *O*. The points $H_{0}$ to $H_{n+2}$ are set on the circumference of *O* at angular distance θ, with $H_{0}$ opposite of *A* in respect to *O* and $H_{i}$ rotating clockwise. Connecting $H_{1}$ to $H_{n+2}$ with *A*, creates the points $F_{1}$ to $F_{n+2}$ where they intersect line M−N at the angles $\phi_{1}$ to $\phi_{n+2}$ (Figure 3, step 1–5). Each set of points $H_{i=0}$ and $H_{i+1}$, till $H_{i=n+2}$, is connected along the circumference of *O*. The lines $H_{i=1}-A$ till $H_{i=n+2}-A$ are mirrored across the $H_{i}$ connections, creating the lines $H_{1}-A_{1}^{\prime }$ to $H_{n+2}-A_{n+2}^{\prime }$ and the points $F_{1}^{\prime }$ to $F_{n+2}^{\prime }$. Rotating the lines $H_{i}-A_{i}^{\prime }$ by θ around $H_{i}$ in clockwise direction creates the lines $H_{1}-B_{1}$ to $H_{n+2}-B_{n+1}$. Points $V_{1}$ to $V_{n+1}$ are set at intersections of $H_{i}-B_{i}$ and $F_{i+1}^{\prime }-M_{i+1}^{\prime }$ (Figure 3, step 6–9). $F_{i}^{\prime }$ and $V_{i}$ are adjusted by rotating their connecting line around $F_{i}^{\prime }$ by the angle $\phi_{i}$ to get $V_{i}^{\prime }$ and offsetting $F_{i+1}^{\prime \prime }$ in parallel before deflecting again around $F_{i+1}^{\prime \prime }$ for the next iteration. Finally, the points $A_{i}^{\prime }$ and $B_{i}$ are deflected by $\phi_{i}$ around $F_{i}^{\prime \prime }$ and $V_{i}^{\prime }$ respectively, creating the points $A_{i}^{\prime \prime }$ and $B_{i}^{\prime }$ (Figure 3, step 10–11).

With the intention to build a physical model, the wing model is scaled up, setting $l_{proximal}=l_{distal}=25$ cm, r=5 cm and $\theta =15^{\circ }$ as input parameters for the fold pattern.

**Figure 4:**
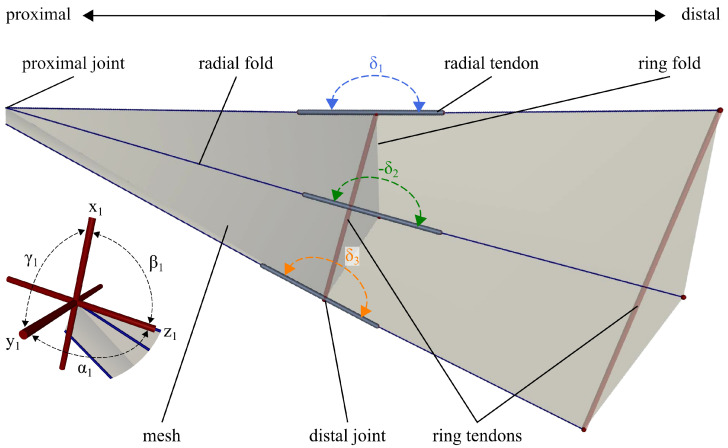
Rendered model of the simulated wing section of one fan folding unit.

Figure 4 shows the model of the wing section created in MuJoCo. The fold lines are modelled as cylindrical bars with an assigned diameter of 0.1 cm and density of $1.25\times 10^{-3}$ kg/cm^3^. A flexible mesh creates the wing membrane in between the bars. The proximal joints have three rotational DoF: $r_{x}$, $r_{y}$ and $r_{z}$, with their angular position $\alpha_{i}$, $\beta_{i}$, and $\gamma_{i}$, with *i* being respective to the number of the joint. The distal joints have one DoF in $r_{x}$, of which the rotational axis is along the ring fold. The distal joint angle $\delta_{i}$ and therefore its range is limited to fold to $180^{\circ }$ and overbend in the open state by $3^{\circ }$. The angular position of each distal joint will be referred to as $\delta_{i}$. The range of every second distal joint is flipped in adaptation to the alternating radial mountain and valley folds present in the anal fan of the dermapteran hindwing. The elasticity of the resilin in the broadened vein patches is modelled as visco-elastic tendons with a variable stiffness *k*. The tendon damping is set constant, at a low value of 0.01 N·cm·s, to help the simulation converge while the initial focus is on the elastic function of the resilin. They are attached to the proximal and distal radial fold lines, spanning around the distal joint. In addition to the stiffness, the resting length $l_{r}$ of the tendons can be adjusted to model a more realistic behaviour of elastomers. The tendons exert a tensional force while $l>l_{r}$, storing elastic energy in the unfolded state and releasing this energy to fold. The simulated tendons can contract below their resting length, such as resilin, which is compressible [18,19]. However, for the initial parameter search, no force, thus no energy input, is required to compress the spatial tendons below their resting length. In addition, two tendons span the ring fold and run parallel to it at the distal end. These tendons have no stiffness and therefore exert no force. Their purpose is to maintain the correct folding configuration and ensure the model’s ability to unfold, whereas this simulator does not consider contact forces.

The wing can be actuated by applying a torque to the proximal and distal joints in $r_{x}$, each in alternating direction for every second joint. The folding process is started by applying only torque on the proximal joints to fold the radial fold, mimicking the force transmission of the basal vein coupling. The motion of the proximal joints initiates the contraction of the tendons, which in turn actuate the folding of the ring fold, when the distal joints align to face in the same direction. To unfold the ring fold, torque is applied directly to the distal joints, substituting the external force when the wing is pulled open by the cerci. The torque on the proximal joints is used to unfold the radial fold. Three states are distinguishable, which are the initial flat state (*a*), the folded state (*b*), and the opened state (*c*). The energy input to transition between the state *a* and *b* is considered as follows:

(3) $$E_{ab}=10^{-2}\cdot \sum_{i=1}^{2n+1}{\left(-1\right)}^{i+1}M_{\alpha }\cdot \Delta \alpha_{i}$$

where $E_{ab}$ is the product of the applied magnitude of torque $M_{\alpha }$, encoding the alternating rotation sign of the joints in accordance with the alternating radial folds by ${\left(-1\right)}^{i+1}$, and the change of the angular position $\Delta \alpha_{i}$ in $r_{x}$ for each actuated joint. Just as the joint rotation is alternating, the joint limit is flipped in sign too. However, the mapping of joint limits to the wing anatomy does not influence the energy. It is to be noted that all information on torque is provided in Ncm and energy in Joules, converting torque from Ncm to Nm. Accordingly, the energy $E_{bc}$ used to open the wing is calculated as follows:

(4) $$E_{bc}=10^{-2}\cdot \sum_{i=1}^{2n+1}\left({\left(-1\right)}^{i}M_{\alpha }\cdot \Delta \alpha_{i}+{\left(-1\right)}^{i+1}M_{\delta }\cdot \Delta \delta_{i}\right)$$

by furthermore adding proximal energy input, as the product of the applied proximal torque $M_{\delta }$, again considering the respective direction as ${\left(-1\right)}^{i+1}$, and the change of angular position $\Delta \delta_{i}$ of the respective joint. Moreover, for a single tendon, the energy *U* at any timestep *t* is defined as follows:

(5) $$U_{t}=10^{-2}\cdot \frac{1}{2}\cdot k\cdot \Delta l^{2}=10^{-2}\cdot \frac{1}{2}\cdot k\cdot {\left(l_{t}-l_{r}\right)}^{2}$$

so that $U_{t}$ is calculated as half of the product of the tendon stiffness *k* and Δl squared, with Δl as the current length $l_{t}$ subtracted by the resting length $l_{r}$. Thereby, *t* can be calculated as the timestep for the respective states *a*, *b*, and *c*.

First, the behaviour of one single tendon is characterised by plotting the energy of the second tendon $U_{t}$ over the deformation of the joint $\delta_{2}$. Thereby, the change of the energy landscape is analysed in dependence on changing values for stiffness and resting length. The selected range for the tendon stiffness for the given wing model is 10–60 N/cm, with an increment of 10 N/cm. Starting from an initial tendon length $l_{i}$ of approximately 11 cm, the range of 0–9 cm is selected for resting lengths, with an increment of 1 cm.

The analysis of a single tendon is followed by the analysis of the whole wing section. The general folding and unfolding process is traceable via the distal joint angles $\delta_{1}$–$\delta_{3}$. This process will be elaborated for one exemplary model of the parameter search, showing the energy landscape of all three tendons in the model. Furthermore, the simulation tool’s numerical stability is analysed by example of this model. Therefore, a timestep sensitivity analysis is conducted, decreasing the timestep width *h* down to 0.003 s at increments of 0.0005 s. For the parameter search, the range of actuating torques is narrowed down incrementally, in steps of 10 Ncm, to identify the feasibility window for successful folding and unfolding and circumstances of failure, for varying combinations of *k* and $l_{r}$. The range is furthermore explored to identify the minimum input energies required to transition between states, a key parameter that is relevant to both the biological organism and technical applications. However, to analyse the unfolding independent of the preceding folding process, a high torque of 500 Ncm is applied to the proximal joints to ensure successful and rapid folding of the wing section. The exact ranges and increments for the parameter search and timestep sensitivity study are summarised in Table 1, which also shows the final, analysed ranges for the applied torques determined during the parameter search.

The simulation results are investigated in regard to whether the identified ranges of *k* and $l_{r}$ are reasonable and support the use of the tool to assist and accelerate the design process of biomimetic, foldable structures inspired by the dermapteran hindwing. For this purpose, a joint is designed and manufactured with multi-material fused deposition modelling, using polyethylene terephthalate glycol (PETG) as a stiff material and thermoplastic urethane (TPU) 82A as an elastic material for the tendon. In a rapid prototyping process, the joint is optimised to the point where specific characteristics meet the technical requirements. Thereby, the overarching goal is to replicate the bistable behaviour, while considering the tendon’s initial length, resting length, and stiffness by means of the simulation results. The joint is to be designed as a print-in-place hinge with one rotational DoF, with bars and hinge structures being stiff enough to withstand the tendon’s forces without breaking. As the qualitative requirements for the bistable joint are met, the material of the elastic tendon is characterised by conducting cyclic tensile tests and compared to the simulation. The aim is to quantify the real, non-linear material behaviour and compare it with the ideal elastic behaviour from the simulations. Therefore, the ZwickRoell zwickiLine 2.5 kN is used together with the software TextXpert II v3.5. In total, five samples of the tendon are pulled to a 100% elongation five times, using a testing speed of 10 mm/min. The test results contain the elongation with the respective force, which are used to plot the strain-stress curve, whereas the strain ε is calculated as follows:

(6) $$\varepsilon =\frac{\Delta l}{l_{0}}$$

which is the elongation Δl divided by the initial length $l_{0}=60$ mm. Furthermore, the stress σ is defined as follows:

(7) $$\sigma =\frac{F}{A_{0}}$$

where the force *F* divided by the initial cross-sectional area $A_{0}=6$ mm^2^. Ultimately, the Young’s modulus *E* can be calculated as follows:

(8) $$E=\frac{\sigma }{\varepsilon }=\frac{F\cdot l_{0}}{A_{0}\cdot \Delta l}$$

Due to non-linearities of the material, such as plastic deformation and the Mullins effect, the first cycle is not considered. For the remaining cycles, the distribution of the Young’s modulus at any measuring point is investigated. Therefore, data is classified by strain, using increments of 10%, allowing for the characterisation of the material’s behaviour locally within the loading and unloading cycles.

## 3. Results and Discussion

### 3.1. Single Tendon

**Figure 5:**
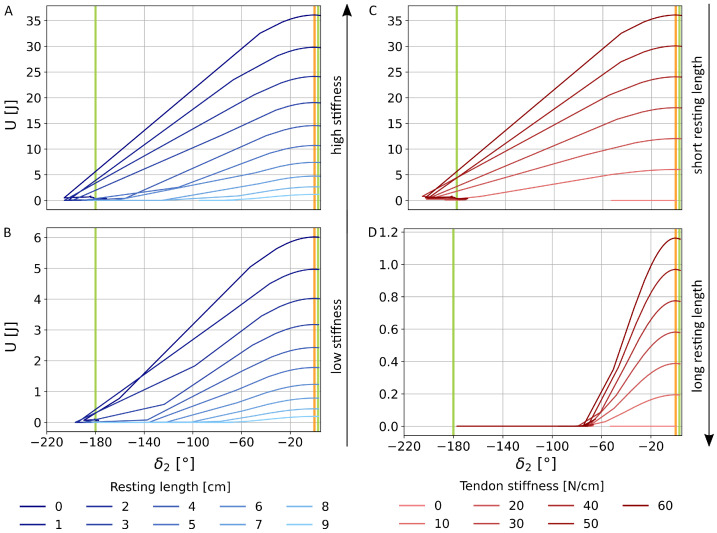
Influence of different resting lengths on the energy landscape for tendons with a high stiffness, k=60 Ncm (**A**) and low stiffness, k=10 N/cm (**B**), and the influence of the tendon stiffness on tendons with a short resting length, $l_{r}=0$ cm (**C**) and long resting length, $l_{r}=9$ cm (**D**). The energy landscape is depicted for the second tendon and distal joint, transitioning from the unfolded to folded state observable by the increasing fold angle. The energetic minima are indicated by the green vertical lines, and the energetic barrier is marked with an orange vertical line.

To understand the bistable behaviour of a wing section, it is important to investigate the behaviour of a single tendon spanning one distal joint. This is done as a function of the design parameters, which are, as aforementioned, the stiffness *k* and resting length $l_{r}$ of the tendon. Figure 5 showcases the change of the energetic landscape depending on the design parameters obtained through the parameter search. The flat and folded state of the joints are two stable positions, given as the two local minima at 3° and approximately −180° respectively, separated by the energy barrier at $0^{\circ }$. This barrier is formed by the overbending allowance of $3^{\circ }$. Due to the compliance of the solver and missing contact forces, it is possible that the joint angle temporarily exceeds the joint limit of $\pm 180^{\circ }$, and oscillates around the point where the lower local energetic minimum lies at the folded state. In consequence, after the folded state is reached, the wing can penetrate itself, allowing the tendons to elongate again. With contact forces, the minima are expected to be reached without overshooting or oscillation. However, implementing contact forces would introduce a significant slowdown of the simulator.

In this system, the unfolded state is the energetically higher, as folding is primarily driven by the release of the tendon energy. Both the absolute potential energy and the energy barrier height increase with higher tendon stiffness and shorter resting length. For example, in the case of a high tendon stiffness and short resting length, like k=60 N/cm and $l_{r}=0$ cm, the potential energy $U_{a}$ exceeds 35 J (Figure 5A). On the contrary, the potential energy $U_{a}$ can lie below 0.2 J, at k=10 N/cm and $l_{r}=9$ cm (Figure 5B).

At lower stiffnesses, the energy barrier at $=0^{\circ }$ is lower and inertia alone is sufficient to complete folding, while no force is exerted by the tendon at lengths below $l_{r}$. This is particularly visible in Figure 5C,D, showcasing the folding for a range of stiffnesses at $l_{r}=0$ cm and $l_{r}=9$ cm. However, the joint does not fold when k=0 N/cm. Due to the absence of elastic properties and therefore potential energy, a transition between states is not possible when the folding is only initiated by the rotation of the proximal joints. This underlines how reliant this bistable system with passive folding is on elastic strain. On the one hand, elastic strain enables the system in the first place. On the other hand, too high strain could also lead to tearing of a physical model or even the biological wing.

The system, here composed of an elastic tendon spanning a joint, can be classified as an asymmetric bistable system that tends toward the folded state. The analysis of the tendon’s mechanical behaviour shows that its potential energy is defined by *k* and $l_{r}$. The two parameters form a trade-off between foldability and stability. Resulting in the initial potential energy $U_{a}$, combinations characterised by high tendon stiffness and short resting lengths increase the stability of the system, to the point where folding is prevented altogether. Conversely, lower stiffnesses facilitate foldability at the cost of stability, as the established energy barrier is lower. However, sufficient energy must be provided to unfold and lock the joint. In conclusion, the tendon’s mechanical behaviour indicates a compromise between the analysed parameters, defining an optimal window to fold and unfold an entire wing section. Thereby, these transitions need to remain feasible while structural locking is preserved. A wing section contains three tendons, which are either stretched when open or contracted when folded. Each tendon, for example, with a stiffness of 10 N/cm and a resting length of 0 cm, has an approximate potential energy of 6 J. Considering the three tendons independently, the theoretical energy landscape holds local minima at 18 J, 12 J, 6 J, or 0 J for the three tendons as a whole, depending on whether none, one, two, or three joints are folded. This theoretical landscape, however, does not account for the interdependence of the joint movements. Thus, the coupled system of the wing section must be evaluated to determine whether the lowest energetic state, the full fold, is achievable.

### 3.2. Wing Section

**Figure 6:**
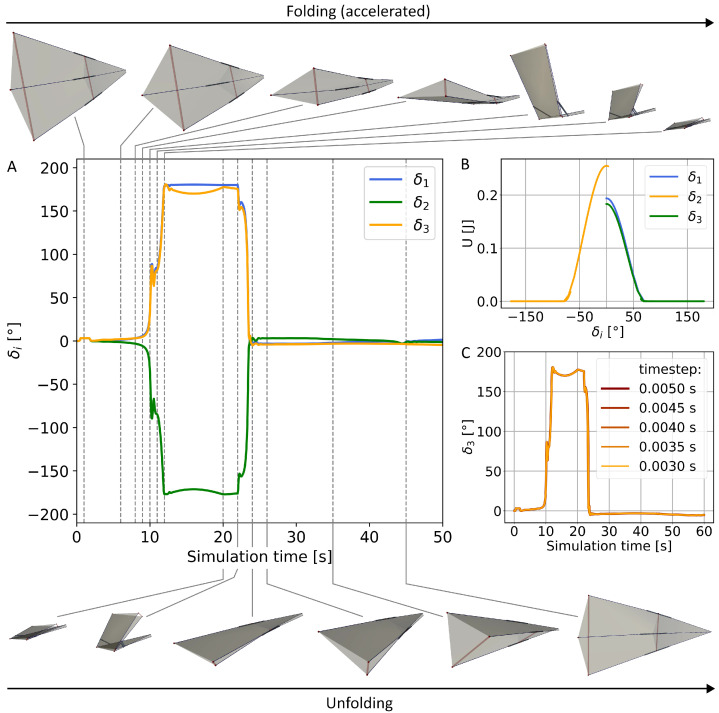
Visualisation of the folding and unfolding process for the model as the joint angles plotted over the simulation time, with the respective frames out of the simulation video (**A**). For this model, a torque of 500 Ncm on the proximal joints is used to accelerate folding, while 20 Ncm of torque on the distal joints is used for unfolding. The energy landscape of all three tendons showcases the locking through the structural asymmetry (**B**). Based on this model, the timestep sensitivity analysis was performed, and the results are plotted over time for the different timesteps using the folding angle of the third joint (**C**).

Building on this, the mechanical behaviour of a whole wing section is now examined to determine the parameter space that enables robust folding and unfolding. With the selected actuation approach, the lowest minimum of the theoretical energy landscape is reached, confirming that a full fold is achievable in practice and is furthermore reversible, returning back into a stable, unfolded state. The folding and unfolding process, as well as the results of the timestep sensitivity analysis, are illustrated using an exemplary model, with 10 N/cm tendon stiffness and 9 cm resting length, from the simulation series in Figure 6.

The wing section of one unit contains three radial struts. In the flat state, all three tendons contract simultaneously, pulling all joints in the positive direction, to a 3° position (Figure 6A). This results in a cambered wing model with mechanical locking: the second joint is counteracted by the first and third, establishing the energy barrier through structural asymmetry (Figure 6B).

Through this approach, the dual stiffness of the hindwing fan is achieved, characterised by the stiff open wing section and flexibility for folding. Driven by the proximal torque and inertial effects, the wing section folds first along the radial fold line, unlocking the wing in turn, and then folds along the ring fold due to the tendon force. In the unfolding process, the ring fold opens before the radial fold line, which is supported by the distal joints’ actuation in addition to proximal joints (Figure 6A). Whereas the folding and unfolding of the radial folds and the ring fold appear simultaneous in the insect wing [3,4], their folding appears rather sequentially in the upscaled model. Due to higher mass, it is expected that actuating torques need to be increased, greater stiffnesses of elastic components are required, and the folding process is slowed down. The upscaled model is not intended to satisfy strict biological scaling. While geometric similarity is preserved, particularly the folding angles required for flat-foldability, the selected densities and mass distributions are chosen to ensure the physical prototype’s feasibility. Dynamic similarity is therefore not imposed, and parameters such as mass, stiffness and actuating torques are not expected to scale proportionally to the insect wing. Despite these differences, the global folding, though slower and sequential, still exhibits bistable behaviour driven by stored elastic energy, while deliberately decoupling it from insect-scale magnitudes. The change of the distal folding angle reveals a rapid transition between the folded and unfolded state, consistent with the abrupt angular movement of the radiating and intercalary veins at the broad vein patches reported in Haas (1994) and Haas and Wootton (1996) [4,13].

By example of this model, the joint angle $\delta_{3}$ of the third distal joint is plotted in Figure 6C for five simulations using incrementally decreased timestep widths. The general output of the simulation does not change in consequence. The course of the folding angle shows negligible variation, and characteristic events, like the sudden folding and unfolding, happen at the same time during the simulation. The oscillatory behaviour remained unaffected by decreasing timestep widths, indicating the oscillations are of physical, not numerical, matter. This suggests numerical stability and convergence with respect to the temporal discretisation.

**Figure 7:**
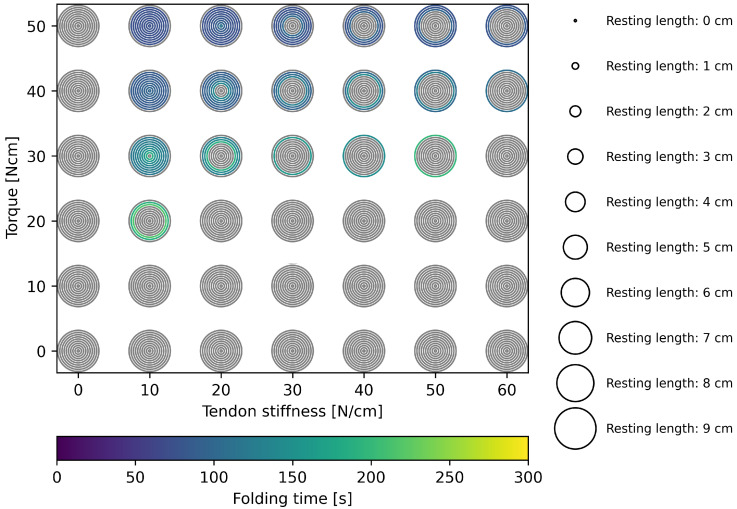
Folding time for the simulated ranges of tendon stiffness, resting length and actuating proximal torque.

In the parameter search, the amount of proximal torque to fold is narrowed down to a range of 0–50 Ncm, at increments of 10 Ncm. To unfold, the range of torque applied on both distal and proximal joints is analysed for up to 100 Ncm. By actuating directly in the distal joints, the unfolding of the ring fold is initiated instantly. However, as the folding is initiated by proximal torque, the folding of the ring fold is delayed until the radial fold is folded. To minimise the energy input, smaller torques can be applied to fold at the cost of the folding time. The lower the torque and the longer the resting length, the more time is required to fold (Figure 7). The minimal torque for successful folding is 20 Ncm for a tendon stiffness of 10 N/cm. In combination with a resting length of 7 cm, this results in 217.45 s to fold. Within the simulated range, the fastest folding, taking 73.95 s, is achieved at 10 N/cm tendon stiffness, 0 cm resting length, using a torque of 50 Ncm.

**Figure 8:**
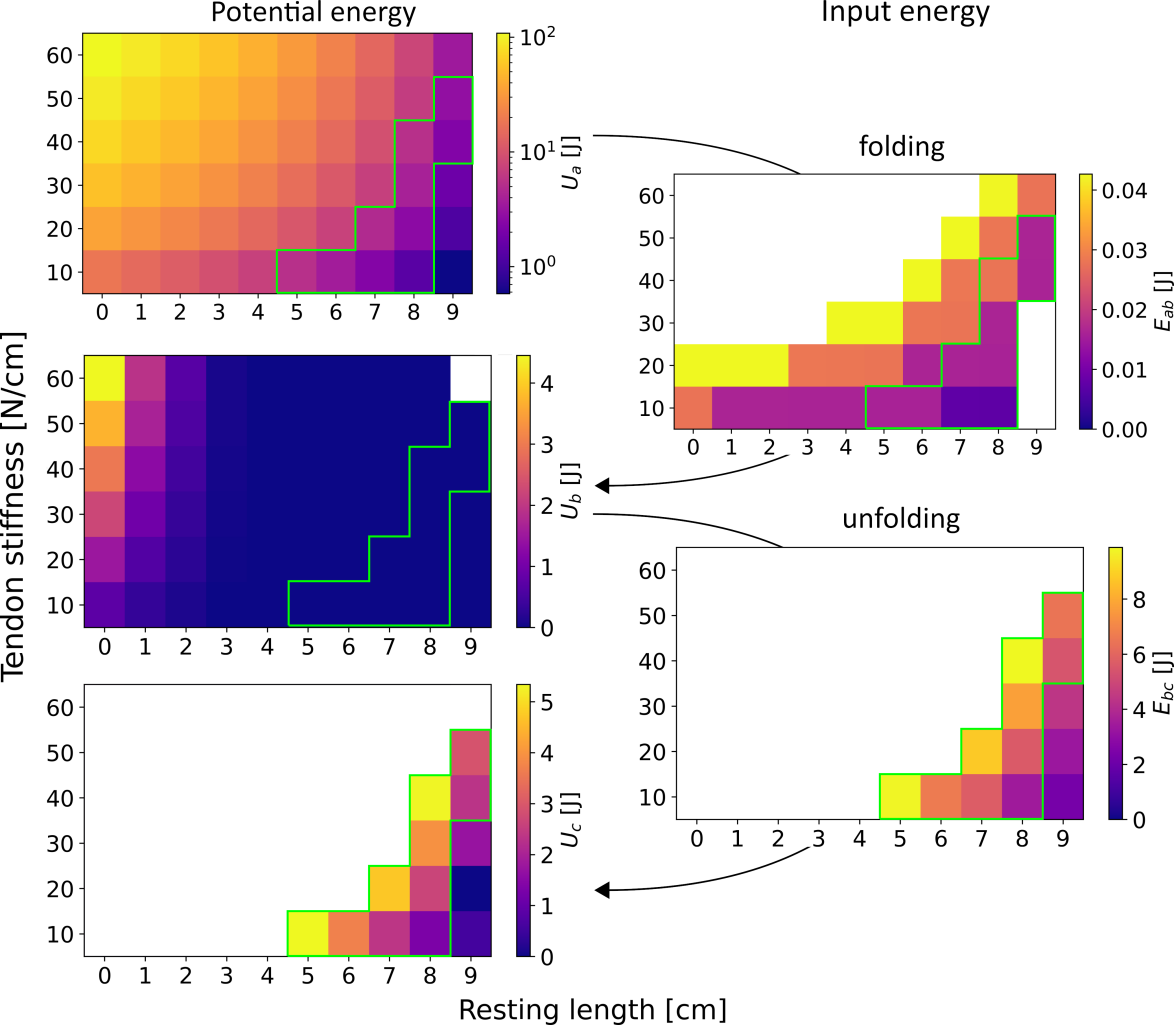
Potential energies of the tendons in the wing section while passing through the different states of flat ($U_{a}$), folded ($U_{b}$), and opened ($U_{c}$), and the respective input energies to make the transitions: $E_{ab}$ and $E_{bc}$. The green border depicts the boundary of physical feasibility.

Similarly to the folding time, both the potential energies of the wing section in all states and the input energies required for the transitions increase with higher tendon stiffness *k* and shorter resting lengths $l_{r}$ (Figure 8). It has to be noted that the exact values are sensitive to the exact time-step *t* selected for the calculation of the potential energy of the tendons $U_{t}$.

Reflecting the behaviour of a single joint and tendon, the potential energy of the wing section in the flat state $U_{a}$ is significantly greater than in the opened state $U_{c}$, which in turn is greater than in the folded state $U_{b}$. Furthermore, significantly less input energy is required for folding than for unfolding, so $E_{ab}<<E_{bc}$, as the transition to the folded state uses the energy of the tendons. In most cases, this energy is released entirely during this transition. However, as no contact forces are modelled, the wing can self-penetrate, leading to oscillations in the folded state and thus to residual tendon elongation, so $l_{t}\neq 0$ cm. Consequently, the energy in the folded state $U_{b}\neq 0$ J in the considered timestep *t*, which is particularly visible for short resting lengths, where $l_{r}\leq 2$ cm. As aforementioned in the context of the energy landscape of a single tendon, the residual energy in the folded state is higher, while actually expected to be 0 J, due to the modelling artefact.

Within the tested torque range, 32 of 60 combinations of *k* and $l_{r}$ fold successfully. The overall minimum is approximately 0.002 J for cases with low tendon stiffness of 10 N/cm for resting lengths around 7 cm to 8 cm. However, at resting lengths of 9 cm, it is apparent again that the provided input energy and energy of the tendon are not sufficient to fold the wing section, given a tendon stiffness equal to or lower than 30 N/cm. This forms a lower limit at which the wing folding is not successful within the tested range of parameters. On the contrary, the upper limit is formed by combinations of high stiffnesses with short resting lengths, which keep the wing section in its flat state and sufficiently counteract the initiating torque. Within the cases that do fold, the maximum amount of input energy $E_{ab}$ is around 0.0042 J, at parameter combinations like $l_{r}=0$ cm and k=20 N/cm or $l_{r}=8$ cm and k=60 N/cm, among others. In total, the upper and lower limit form a boundary window for mechanical feasibility, which is further narrowed when trying to unfold the wing section.

For successful unfolding, the provided input energy must exceed the potential energy of the wing section in the unfolded state, so $E_{bc}>U_{A}$ must be given. This is to be expected given the asymmetric energy landscape, as both energy and torque are required to overcome the tendon’s stiffness. It has to be noted that the combination of $l_{r}=9$ cm and k=60 N/cm does not fold completely when applying a proximal torque of 500 Ncm, despite fully folding when using lower torques. The distal joints of the wing section get stuck at $\pm 75^{\circ }$. However, the initial folding behaviour is comparable to that of both shorter resting lengths and lower stiffnesses, leading us to attribute this outlier to numerical imprecision. A numerical error in the integration of the joint positions and velocities, likely caused by this combination of parameters, leads to the solution converging at the previously mentioned angle of the distal joints. In total, only 13 cases of the tested parameter combinations complete the transition from folded to unfolded. Unfolding is most successful for combinations of long resting lengths and low stiffnesses. Given that, the minimum input energy $E_{bc}$ is to be found at $l_{r}=9$ cm and k=10 N/cm with 1.898 J. The maximum is 8.056 J at $l_{r}=5$ cm and k=5 N/cm.

All in all, the results demonstrate how structural asymmetry creates a mechanical lock in the flat state, while asymmetric bistability determines the energetic cost of transitions. Folding is energetically cheap and easier to achieve, whereas unfolding is restricted to a narrower parameter space and requires significantly higher input energy. The simulation depicts a boundary separating the parameter space for successful and unsuccessful folding and unfolding transitions. This suggests that the mechanics of biomimetic models, inspired by the dermapteran hindwing, need to be finely tuned to resist unwanted collapse while still permitting controlled folding and unfolding under the right actuation.

### 3.3. Physical Model Feasibility

**Figure 9:**
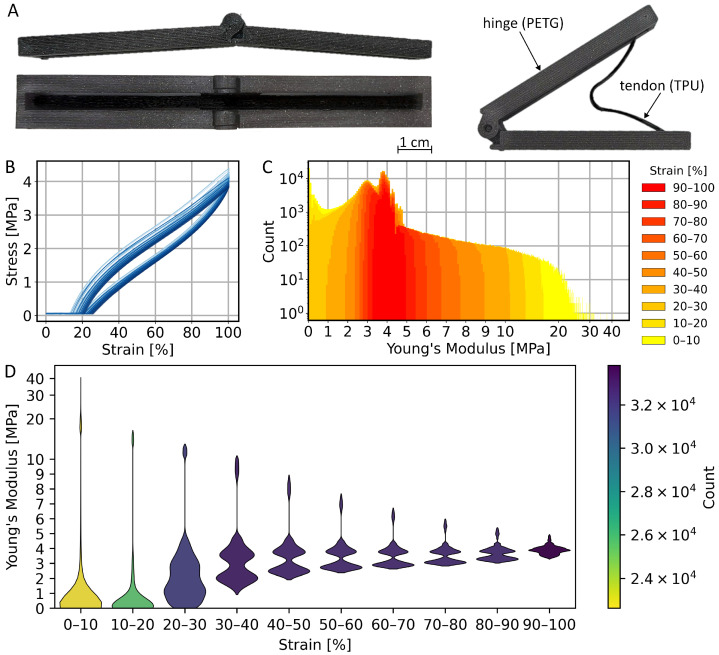
Prototype of a bistable joint (**A**) and results from cyclic testing of the TPU tendon, as a strain-stress curve of five samples, each excluding the first cycle (**B**). The derived Young’s Modulus at each measuring point is depicted as a histogram (**C**) and violin plot (**D**), displaying the absolute and relative distribution for ranges of strain.

To assess the feasibility with accessible materials and manufacturing methods, a prototype was designed based on one single joint of the simulation model. For the simulation model, the overarching qualitative bistable behaviour is consistent across the simulated range of stiffnesses and resting lengths. Therefore, the aim was to replicate the characteristic behaviour and achieve asymmetric bistability within identified boundaries rather than precise parameter matching. Hence, the derived conceptual focus is on recreating the two stable states, open and folded, separated by an energy barrier. This is done by combining a visco-elastic tendon with a one DoF hinge (Figure 9A).

The prototype successfully displayed the qualitative behaviour of a single, simulated distal joint. To establish the energy barrier, the full rotation of the joint is structurally hindered by design, and slight overbending is allowed. The joint’s tendon is made of TPU, selected as a low-cost, readily available material. Comparing the tendon’s parameters with those of the simulation, the printed resting length is 6 cm and the average stiffness is measured to be approximately 4 N/cm at a cross-sectional area of 5 mm^2^. In order to match the simulated stiffnesses, ranging from 10 N/cm to 60 N/cm, the cross-sectional area of the tendon would have to be at least 12.5 mm^2^ up to 75 mm^2^. To ensure the overall stability of the joint design in withstanding the tendon’s force, the tendon design is adjusted for less stiffness and a rather long resting length. The tendon pulls the joint into the folded state, while it locks it in the overbend, open state and therefore establishes the asymmetric bistable behaviour. By applying a small external force, the energy barrier can be overcome to transition from the open to the folded state. Conversely, high force is necessary to counteract the tendon’s force and transition back to the open state. This demonstrates that an asymmetric bistable response and dual stiffness can be achieved, even with simple geometries, inexpensive materials, and accessible manufacturing methods.

While the mechanistic principle of the bistable joint is successfully replicated, several differences emerged on the quantitative side when comparing the physical tendon to the simulation model. As already indicated above, the stiffness is reduced to ensure the qualitative behaviour of the prototype, while maintaining a compact design. Alternatively, a stronger hinge material than PETG can be used in the future to withstand the tendon’s force without breaking. A general redesign can prevent stability issues by distributing the force flow differently across the rigid structures. The physical tendon does not contract further below its resting length. In consequence, the tendon is physically preventing the hinge from fully folding as the tendon gets stuck between the hinge arms, even though the provided tendon energy and inertial effects are expected to be sufficient for folding. However, the cyclic tests revealed the non-linear stress-strain relationship, typical for visco-elastic materials (Figure 9B). Due to creep, drift, and the Mullins Effect, thus softening of the tendon, the tendon’s actual resting length is increasing. The results of the cyclic loading test show that force is only exerted until 35% strain is reached during the unloading cycle. In consequence, the potential energy of the tendon decreases after each cycle and reduces the consistency of the folding performance.

To generalise the results, the properties of the physical tendon are reframed in terms of Young’s modulus rather than stiffness, decoupling the mechanical response from the cross-sectional geometry (Figure 9C,D). The TPU exhibits a broader Young’s modulus range, up to 40 MPa compared to resilin in the insect cuticle of up to 1.5 MPa [21]. However, due to the upscaled geometry of the demonstrator, the absolute moduli do not map directly, and the TPU is therefore used to reproduce the qualitative bistable behaviour rather than to match the stiffness at the insect scale.

Due to the material’s non-linear behaviour, evident in hysteresis and drift, the ranges of Young’s modulus overlapping with the simulated parameter space are also traversed in the process of loading and unloading the tendon, thus during unfolding or folding the joint. As a result, the fine-tuning of the tendons’ stiffness and visco-elastic behaviour toward simulated ranges is, on the one hand, achievable through the choice of material and dimensioning of the tendon geometry, particularly its cross-sectional area and length. On the other hand, the tendons are currently simulated as linear visco-elastic tendon behaviour, with low damping. The tendon behaviour can be modelled closer to realistic, visco-elastic material behaviour, by implementing stiffness and damping as nonlinear functions of tendon length and velocity. This can be done using visco-elastic models like Kelvin–Voigt- or Maxwell-type formulations, of which the latter is common to characterise the visco-elastic response of resilin [56,57]. With such models, the visco-elastic response can be adjusted either to reproduce the biological behaviour more closely or to capture the non-linear stress-strain relationship observed in the TPU. This leads to increased complexity and accuracy in predicting technological transfer compared to the current simplified approach focused on storing elastic energy. The design space for complex bistable structures remains open and practically achievable with improved, upscaled prototypes and closer design assistance by the simulation tool. In summary, the functioning of a simulated tendon and hinge, and furthermore the broad vein patches, are sufficiently captured in the physical prototype by means of storing elastic energy for folding. The prototype demonstrates the feasibility of the approach and provides valuable insights for refining the software and design toward a functional wing-section prototype and advancing the technology readiness level in the future.

## 4. Conclusions

In this work, a simulation tool, available on Gitlab, was developed. The results of an initial parameter search to capture the dynamics of the dermapteran hindwing folding and unfolding were presented to demonstrate the use of the tool. The model, configured with visco-elastic tendons, successfully reproduced the asymmetric bistability and dual-stiffness—two defining properties of the biological model that emerge from the interplay of the folding pattern and material distribution. The system is successfully digitally replicated, capturing the current understanding of the dynamics of the fan folding to the fullest and demonstrating how local elasticities drive global deformation with minimal energy expenditure. The simulations reveal how finely tuned the mechanical properties of the dermapteran hindwing are in order for folding and unfolding to work, as there are clear boundaries to the functionality of a delicately balanced folding system.

With the presented MuJoCo-based approach, a new modelling method for foldable systems is established. These systems can be explored across a wide range of design parameters, bridging the gap in complexity between rigid origami models and FE simulations, where modelling insect wing folding spans from a high degree of simplification to the impossibility of achieving the biological accuracy of complex folding. The simulation tool provides a computationally cheap and fast alternative while being physically meaningful, as a large scope of parameters can be explored, including geometry, mass, and material properties. As a result, the design criteria for successful transitions between the folded and unfolded states become quantifiable, demonstrating transferability and feasibility. The tool accelerates the parameter search for engineered, physical solutions by drastically reducing the need for trial-and-error in prototyping.

The folding processes are currently simulated linearly, with non-linearities of the visco-elastic material for the tendons and the contact forces not yet integrated. In the future, the tool can be further developed to overcome the current limitations and ultimately simulate real folding behaviour with higher accuracy. Together with the physical prototype, which qualitatively reproduced the bistable characteristic of the tendon-joint-architecture within accessible material and manufacturing constraints, the tool provided complementary insights that guide future refinement toward self-stabilising deployable structures with minimal energy input. However, the tool and design parameters are not only adjustable towards engineering applications, but also towards biological research, fine-tuning the visco-elastic tendon’s characteristics toward more resilin-like behaviour, considering, for example, the protein’s Young’s modulus, anisotropy, and compressibility. Additional mechanisms of the dermapteran hindwing can be included, like the mid-wing mechanism, basal vein coupling or the claval flexion line. This paves the way for a comprehensive understanding of the wing with the help of a coherent simulation tool, whereas biomechanical research on such delicate structures is inherently challenging. Instead, the tool enables the analysis of the entire biological folding system with geometry, kinematics, and dynamics proportionally scaled to the dermapteran hindwing, providing an in-depth dimensionless analysis on one hand. On the other hand, the base is set for heuristic testing of hypotheses about evolutionary optimisation, uncovering how close the system operates near failure and which mechanisms are redundant or non-contributory.

In turn, through enhanced understanding of the biological folding system on how local elasticities need to be tailored, and how to efficiently actuate deformation, engineering applications can directly benefit from further development of the tool. This highlights the work’s broader relevance of modelling insect wing folding as a dynamic, scalable system, as it is expanding the biomimetic potential. More comprehensive design principles can be derived for the development of biomimetic, programmable folding structures, informing applications in soft-robotics, medical technology, emergency infrastructure, and aerospace engineering, wherever ultra-lightweight, reconfigurable, and resilient folding systems are essential.

## Figures and Tables

**Table 1:** Ranges and increments studied in the parameter search and timestep sensitivity analysis.

Parameter	Range	Increment
Parameter search		
Tendon stiffness [N/cm]	0–60	10
Resting length [cm]	0–9	1
Torque to fold [Ncm]	0–50	10
Torque for accelerated folding [Ncm]	500	const.
Torque to unfold [Ncm]	0–100	10
Timestep width [s]	0.005	const.
Timestep sensitivity analysis		
Timestep width [s]	0.0030–0.0050	0.0005

## Data Availability

The source code used to run the simulations and the results obtained can be found at https://gitlab.com/EuropeanSpaceAgency/earwig_bistable/-/tree/simulation_tool_v1, accessed on 3 November 2025.

## References

[1] Haas F., Gorb S., Wootton R.J. (2000). Elastic joints in dermapteran hind wings: Materials and wing folding. Arthropod Struct. Dev..

[2] Deiters J., Kowalczyk W., Seidl T. (2016). Simultaneous optimisation of earwig hindwings for flight and folding. Bio. Open.

[3] Kleinow W. (1966). Untersuchungen zum Flügelmechanismus der Dermapteren. Z. Morphol. Oekol. Tiere..

[4] Haas F. (1994). Geometry and Mechanics of Hind-Wing Folding in Dermaptera and Coleoptera. Ph.D. Thesis.

[5] Wiedemann J. (2007). Leichtbau: Elemente und Konstruktion.

[6] Yue S. (2023). A Review of Origami-Based Deployable Structures in Aerospace Engineering. J. Phys. Conf. Ser..

[7] Liu T.-W., Bai J.-B., Fantuzzi N., Zhang X. (2024). Thin-walled deployable composite structures: A review. Prog. Aerosp. Sci..

[8] Zander M., Belvin W.K. Concept-development of a structure supported membrane for deployable space applications-from nature to manufacture and testing. Proceedings of the European Conference on Spacecraft Structures, Materials and Environmental Testing, 2012.

[9] Zander M.E., Chamberlain M.K., Jost D., Müller D.R., Hagmeister N., Straubel M., Hühne C. Design and testing of the BionicWingSat in a zero-g flight campaign-a 2U-CubeSat with deployable, biologically-inspired wings. Proceedings of the AIAA SciTech 2023 Forum.

[10] Binder N., Hofmann L., Killian M., Stelzl D., Seidl T. Falten, Öffnen, Stabilisieren: Wie Bionik Satellitenbremssegel verbessern kann. Proceedings of the 10. Bionik-Kongress 2023.

[11] Chen Y., Peng R., You Z. (2015). Origami of thick panels. Science.

[12] Luo Q., Wei F., Liu W. (2025). Foldable insect wings: From folding and unfolding mechanisms to inspired applications. J. Exp. Biol..

[13] Haas F., Wootton R.J. (1996). Two basic mechanisms in insect wing folding. Proc. R. Soc. Lond. B.

[14] Wang C., Wang C., Ning Y., Chen L., Wang X. (2018). Design and Mechanical Analysis of Bionic Foldable Beetle Wings. Appl. Bionics Biomech..

[15] Cao Y., Derakhshani M., Fang Y., Huang G., Cao C. (2021). Bistable Structures for Advanced Functional Systems. Adv. Funct. Mater..

[16] Faber J.A., Arrieta A.F., Studart A.R. (2018). Bioinspired spring origami. Science.

[17] Elvin C.M., Carr A.G., Huson M.G., Maxwell J.M., Pearson R.D., Vuocolo T., Liyou N.E., Wong D.C.C., Merritt D.J., Dixon N.E. (2005). Synthesis and properties of crosslinked recombinant pro-resilin. Nature.

[18] Weis-Fogh T. (1961). Molecular interpretation of the elasticity of resilin, a rubber-like protein. J. Mol. Biol..

[19] Michels J., Appel E., Gorb S.N., Cohen E., Moussian B. (2016). Resilin—The Pliant Protein. Extracellular Composite Matrices in Arthropods.

[20] Haas F., Gorb S., Blickhan R. (2000). The function of resilin in beetle wings. Proc. R. Soc. Lond. B.

[21] Vincent J.F.V., Wegst U.G.K. (2004). Design and mechanical properties of insect cuticle. Arthropod Struct. Dev..

[22] Anas S.M., Tahzeeb R., Al-Dala’ien R.N., Alam M., Shariq M. (2024). Widely Employed Constitutive Material Models in Abaqus FEA Software Suite for Simulations of Structures and Their Materials: A Brief Review. E3S Web Conf..

[23] Rojas S., Riley K.S., Arrieta A.F. (2022). Multistable bioinspired origami with reprogrammable self-folding. J. R. Soc. Interface.

[24] Han H., Yu B., Tang L., Zhao N., Cao D., Wang Y. (2025). Dynamics of flexible multi-stable origami with bio-inspired creases. Int. J. Mech. Sci..

[25] Saito K., Yamamoto S., Maruyama M., Okabe Y. (2014). Asymmetric hindwing foldings in rove beetles. Proc. Natl. Acad. Sci. USA.

[26] Saito K., Nomura S., Yamamoto S., Niiyama R., Okabe Y. (2017). Investigation of hindwing folding in ladybird beetles by artificial elytron transplantation and microcomputed tomography. Proc. Natl. Acad. Sci. USA.

[27] Saito K., La Pérez-de Fuente R., Arimoto K., Seong Y.A., Aonuma H., Niiyama R., You Z. (2020). Earwig fan designing: Biomimetic and evolutionary biology applications. Proc. Natl. Acad. Sci. USA.

[28] Deiters J., Kowalczyk W., Seidl T. Strukturelle Stabilisierung des Dermapterenflügels. Proceedings of the Bionik: Patente aus der Natur—Tagungsbeiträge zum 7. Bionik-Kongress.

[29] Appel E., Michels J., Gorb S.N. (2024). Resilin in Insect Flight Systems. Adv. Funct. Mater..

[30] Haas F., Kukalová-Peck J. (2001). Dermaptera hindwing structure and folding: New evidence for familial, ordinal and superordinal relationships within Neoptera (Insecta). Eur. J. Entomol..

[31] Haas F., Hwen J.T.C., Tang H. (2012). New evidence on the mechanics of wing unfolding in Dermaptera (Insecta). Arthropod Syst. Phylogeny.

[32] Melis J.M., Siwanowicz I., Dickinson M.H. (2024). Machine learning reveals the control mechanics of an insect wing hinge. Nature.

[33] Ghassaei A., Demaine E.D., Gershenfeld N. (2018). Fast, interactive origami simulation using GPU computation. Origami.

[34] Schenk M., Guest S.D., Yim M. (2011). Origami folding: A Structural Engineering Approach. Origami5.

[35] Zhang T., Kawaguchi K., Wu M. (2018). A folding analysis method for origami based on the frame with kinematic indeterminacy. Int. J. Mech. Sci..

[36] Zhang T., Kawaguchi K. (2021). Folding analysis for thick origami with kinematic frame models concerning gravity. Automat. Constr..

[37] Tachi T. (2009). Simulation of rigid origami. Origami.

[38] Schenk M., Guest S.D., McShane G.J. (2014). Novel stacked folded cores for blast-resistant sandwich beams. Int. J. Solids Struct..

[39] Ali Ablat M., Qattawi A. (2018). Finite Element Analysis of Origami-Based Sheet Metal Folding Process. J. Eng. Mater. Technol..

[40] Liu K., Paulino G.H. MERLIN: A MATLAB implementation to capture highly nonlinear behavior of non-rigid origami. Proceedings of the IASS Annual Symposia.

[41] Filipov E.T., Liu K., Tachi T., Schenk M., Paulino G.H. (2017). Bar and hinge models for scalable analysis of origami. Int. J. Solids Struct..

[42] Zhu Y., Schenk M., Filipov E.T. (2022). A Review on Origami Simulations: From Kinematics, to Mechanics, Toward Multiphysics. Appl. Mech. Rev..

[43] Kitajima C., Saito K., Taiju Y., Nishi K., Suehiro K. (2024). Thickness Accommodation in Earwig Fan Folding. Res. Sq..

[44] Kitajima C., Taiju Y., Nishi K., Sehiro K., Saito K. (2025). Earwig fan folding with thick panels. Bioinspir. Biomim..

[45] Alexander R.M. (2003). Modelling approaches in biomechanics. Phil. Trans. R. Soc. Lond. B Biol. Sci..

[46] Wootton R.J., Herbert R.C., Young P.G., Evans K.E. (2003). Approaches to the structural modelling of insect wings. Phil. Trans. R. Soc. Lond. B Biol. Sci..

[47] Eshghi S., Nooraeefar V., Darvizeh A., Gorb S.N., Rajabi H. (2020). WingMesh: A Matlab-Based Application for Finite Element Modeling of Insect Wings. Insects.

[48] Eshghi S., Nabati F., Shafaghi S., Nooraeefar V., Darvizeh A., Gorb S.N., Rajabi H. (2022). An image based application in Matlab for automated modelling and morphological analysis of insect wings. Sci. Rep..

[49] Jin T., Goo N.S., Park H.C. (2010). Finite Element Modeling of a Beetle Wing. J. Bionic. Eng..

[50] Herbert R.C., Young P.G., Smith C.W., Wootton R.J., Evans K.E. (2000). The hind wing of the desert locust (Schistocerca gregaria Forskål). III. A finite element analysis of a deployable structure. J. Exp. Biol..

[51] Singh B., Ahmad K.A., Murugaiah M., Yidris N., Basri A.A., Pai R. (2024). Quasi-steady aerodynamic modeling and dynamic stability of mosquito-inspired flapping wing pico aerial vehicle. Front. Robot. AI.

[52] (2024). MuJoCo.

[53] Ishiguro R., Kawasetsu T., Motoori Y., Paik J., Hosoda K. (2023). Earwig-inspired foldable origami wing for micro air vehicle gliding. Front. Robot. AI.

[54] (2025). Python.

[55] DeepMind Technologies Limited MuJoCo Documentation: Version 3.2.0. https://mujoco.readthedocs.io/en/3.2.0/.

[56] Kovalev A., Filippov A., Gorb S.N. (2018). Slow viscoelastic response of resilin. J. Comp. Physiol. A.

[57] Filippov A.E., Gorb S.N. (2020). Biomechanics at the Microscale. Combined Discrete and Continual Approaches in Biological Modelling.

